# Dietary Nitrate Supplementation Does Not Alter Exercise Efficiency at High Altitude – Further Results From the Xtreme Alps Study

**DOI:** 10.3389/fphys.2022.827235

**Published:** 2022-02-28

**Authors:** Philip J. Hennis, Andrew F. Cumpstey, Alasdair F. O’Doherty, Bernadette O. Fernandez, Edward T. Gilbert-Kawai, Kay Mitchell, Helen Moyses, Alexandra Cobb, Paula Meale, Helmut Pöhnl, Monty G. Mythen, Michael P. W. Grocott, Denny Z. H. Levett, Daniel S. Martin, Martin Feelisch, The Xtreme Alps Research Group, Adam Booth

**Affiliations:** ^1^Centre for Altitude Space and Extreme Environment Medicine, University College London Hospital NIHR Biomedical Research Centre, Institute of Sport, Exercise and Health, London, United Kingdom; ^2^SHAPE Research Group, School of Science and Technology, Nottingham Trent University, Nottingham, United Kingdom; ^3^Perioperative and Critical Care Theme, NIHR Southampton Biomedical Research Centre, University Hospital Southampton NHS Foundation Trust, Southampton, United Kingdom; ^4^Integrative Physiology and Critical Illness Group, Clinical and Experimental Sciences, Faculty of Medicine, University of Southampton, Southampton, United Kingdom; ^5^Department of Sport, Exercise and Rehabilitation, Northumbria University, Newcastle upon Tyne, United Kingdom; ^6^Division of Metabolic and Vascular Health, Warwick Medical School, University of Warwick, Coventry, United Kingdom; ^7^AURAPA Würzungen GmbH, Bietigheim-Bissingen, Germany; ^8^Peninsula Medical School, University of Plymouth, Plymouth, United Kingdom

**Keywords:** exercise, high altitude, hypoxia, nitric oxide, beetroot, nitrite, nitrate

## Abstract

**Introduction:**

Nitrate supplementation in the form of beetroot juice (BRJ) ingestion has been shown to improve exercise tolerance during acute hypoxia, but its effect on exercise physiology remains unstudied during sustained terrestrial high altitude exposure. We hypothesized that performing exercise at high altitude would lower circulating nitrate and nitrite levels and that BRJ ingestion would reverse this phenomenon while concomitantly improving key determinants of aerobic exercise performance.

**Methods:**

Twenty seven healthy volunteers (21 male) underwent a series of exercise tests at sea level (SL, London, 75 m) and again after 5–8 days at high altitude (HA, Capanna Regina Margherita or “Margherita Hut,” 4,559 m). Using a double-blind protocol, participants were randomized to consume a beetroot/fruit juice beverage (three doses per day) with high levels of nitrate (∼0.18 mmol/kg/day) or a nitrate-depleted placebo (∼11.5 μmoles/kg/day) control drink, from 3 days prior to the exercise trials until completion. Submaximal constant work rate cycle tests were performed to determine exercise efficiency and a maximal incremental ramp exercise test was undertaken to measure aerobic capacity, using breath-by-breath pulmonary gas exchange measurements throughout. Concentrations of nitrate, nitrite and nitrosation products were quantified in plasma samples collected at 5 timepoints during the constant work rate tests. Linear mixed modeling was used to analyze data.

**Results:**

At both SL and HA, plasma nitrate concentrations were elevated in the nitrate supplementation group compared to placebo (*P* < 0.001) but did not change throughout increasing exercise work rate. Delta exercise efficiency was not altered by altitude exposure (*P* = 0.072) or nitrate supplementation (*P* = 0.836). V̇O_2_peak decreased by 24% at high altitude (*P* < 0.001) and was lower in the nitrate-supplemented group at both sea level and high altitude compared to placebo (*P* = 0.041). Dietary nitrate supplementation did not alter other peak exercise variables or oxygen consumption at anaerobic threshold. Circulating nitrite and S-nitrosothiol levels unexpectedly rose in a few individuals right after cessation of exercise at high altitude.

**Conclusion:**

Whilst regularly consumed during an 8 days expedition to terrestrial high altitude, nitrate supplementation did not alter exercise efficiency and other exercise physiological variables, except decreasing V̇O_2_peak. These results and those of others question the practical utility of BRJ consumption during prolonged altitude exposure.

## Introduction

Increasingly, people are traveling to high altitude and performing physical activity for the purposes of work, recreation, and sport. In doing so, these individuals are exposed to atmospheric hypobaric hypoxia, which impedes physical and cognitive performance and can cause the onset of high altitude illness (Hennis et al., 2016; Griva et al., 2017). Performing exercise under hypoxic conditions further increases arterial hypoxemia (Roach et al., 2000), and may predispose for the development of acute mountain sickness (Taylor, 2011). Whilst the mechanisms responsible for altitude-induced reductions in physical and cognitive performance are not fully understood, harmful effects of atmospheric hypoxia appear to be related to impaired oxygen transport and/or utilization pathways. The deleterious effects of atmospheric hypoxia could be mitigated by interventions that target mechanisms central to these pathways.

Nitric oxide (NO) is an important mediator of human physiological responses to hypoxia, not only because of its effects on pulmonary and cardiovascular function, erythropoiesis and metabolic regulation (Beall et al., 2012; Siervo et al., 2014; Luks et al., 2017; Murray et al., 2018) but also due to its ability to match energy supply and demand at the cellular level (Umbrello et al., 2013). Consistent with these actions, high altitude natives have elevated plasma nitrate and nitrite concentrations (biomarkers of NO production) when compared to sea level controls (Erzurum et al., 2007), and lowlanders increase plasma concentrations of NO metabolites (nitrate, nitrite) and cyclic guanosine 3′,5′-monophosphate (cGMP) during the acclimatization process (Janocha et al., 2011; Levett et al., 2011). As such, enhanced NO production is a universal response to hypoxic stress (Feelisch, 2018) and may be advantageous for acclimatizing and exercising at high altitude. A significant part of whole-body NO production occurs via the enzymatic oxidation of L-arginine by the NO synthase (NOS) family of enzymes. An alternative, NOS-independent, pathway to elevate NO production is thought to be provided by increasing the dietary intake of nitrate (Butler and Feelisch, 2008). An alternative, NOS-independent mechanism through which NO may be generated in the body is via the reduction of nitrate. The sequential reduction of nitrate to nitrite and NO via this so-called non-canonical Nitrate-Nitrite-NO pathway has been proposed to be particularly active in hypoxic environments (Lundberg et al., 2008, 2009). Increasing intake of dietary nitrate and elevating plasma nitrate and nitrite levels, with the latter being converted to NO and nitroso species, could have the potential to alter many physiological outcomes (Lundberg et al., 2008). Notable dietary nitrate-induced physiological changes include reduced resting blood pressure (Bahadoran et al., 2017) and improved endurance exercise performance [for reviews see (McMahon et al., 2017; Jones et al., 2018, 2021; Olsson et al., 2019)]. A growing body of evidence, mostly obtained in studies conducted at SL, suggests that dietary nitrate supplementation, via the consumption of nitrate-rich beetroot or green leafy vegetables, could improve key physiological determinants of aerobic exercise performance to enhance hypoxic exercise tolerance.

Administration of beetroot juice (BRJ) has been reported to improve hypoxic time trial performance for running (Shannon et al., 2016), cycling (Muggeridge et al., 2014), walking (Shannon et al., 2017), and to prolong time to exhaustion during cycling (Masschelein et al., 2012; Kelly et al., 2014; Horiuchi et al., 2017) and knee-extension exercise (Vanhatalo et al., 2011). However, in other studies BRJ had no ergogenic effect on running, cycling, or skiing time trial performance (Arnold et al., 2015; MacLeod et al., 2015; Nybäck et al., 2017), nor did it alter time to exhaustion during cycling, walking and forearm exercise (Carriker et al., 2016; Gasier et al., 2017; Rossetti et al., 2017). Any ergogenic effect of BRJ will occur through its action on one or more of the four physiological determinants of aerobic exercise performance; (i) peak oxygen consumption (V̇O_2_peak), (ii) ventilatory anaerobic threshold (AT), (iii) exercise economy, and (iv) oxygen uptake kinetics (Jones et al., 2008). Of these determinants of performance, supplementation with BRJ during hypoxia has been reported to alter exercise economy (Masschelein et al., 2012; Kelly et al., 2014; Muggeridge et al., 2014; Shannon et al., 2016; Nybäck et al., 2017), V̇O_2_peak, and oxygen uptake kinetics (Kelly et al., 2014). The role of BRJ on hypoxic exercise economy is most convincing with decreases in submaximal O_2_ utilization with BRJ compared to a placebo reported for a range of exercise intensities and modalities (Masschelein et al., 2012; Kelly et al., 2014; Muggeridge et al., 2014; Shannon et al., 2016, 2017), although conflicting data exists (MacLeod et al., 2015; Horiuchi et al., 2017; Nybäck et al., 2017; Rossetti et al., 2017). These studies support a body of evidence attesting to a BRJ-induced improvement in the efficiency of oxygen use during exercise in normoxia, which has been suggested to occur due to greater efficiency of ATP resynthesis (i.e., higher mitochondrial P/O ratio) and/or improved muscle contraction efficiency [for reviews see (Affourtit et al., 2015; Jones et al., 2021)]. Hypoxic V̇O_2_peak has been shown to be both lower (Kelly et al., 2014) and unchanged (Masschelein et al., 2012; Arnold et al., 2015; Nybäck et al., 2017) following BRJ administration, whilst the only study regarding V̇O_2_ kinetics reported BRJ to lower Tau (the time taken for oxygen uptake to reach 63% of its final amplitude following a stepwise increase in work rate) during moderate but not severe intensity exercise (Kelly et al., 2014). It is noteworthy that all of the aforementioned studies “simulated” high altitude conditions by acutely exposing participants to normobaric hypoxia. Whilst such studies provide useful contributions to understanding whether BRJ has the potential to ameliorate reductions in performance determinants upon acute exposure, their validity to conditions at high altitude environments (i.e., in hypobaric hypoxia) could be challenged.

The distinction between “simulated” hypoxic and terrestrial altitude exposure is likely important given NO metabolism and physiological acclimation to hypoxia can differ according to whether hypobaric or normobaric hypoxia is employed [for review see, (Coppel et al., 2015)]. Furthermore, none of the “simulated” altitude studies, using either normobaric or hypobaric hypoxia, have investigated the efficacy of BRJ on exercise physiological responses during hypoxic exposure of more than a few hours. Altitude acclimatization is a dynamic process, and thus the impact of BRJ on physiological responses to very acute hypoxia may not translate when the hypoxic dose is delivered over a number of days or weeks. The majority of people who contend with hypoxic conditions do so over a prolonged period of time at terrestrial high altitude; thus, studying the potential ergogenic effect of BRJ on exercise responses in this setting is required to address whether or not BRJ has effects on exercise performance in the field.

We hypothesized that performing physical exercise at high altitude would increase tissue utilization of nitrate and nitrite as a result of the combination of metabolic (working muscle) and environmental (hypobaric) hypoxia, and that dietary nitrate supplementation could reverse this phenomenon by altering key determinants of aerobic exercise performance (particularly exercise efficiency – i.e., the ratio of mechanical work to energy expenditure during exercise) during sustained exposure to terrestrial high altitude.

## Materials and Methods

### Participants

Twenty seven healthy volunteers completed the study [21 male; age, 28.9 (±5.2) years; stature, 177 (±8) cm; body mass, 74.0 (±11.3) kg; V̇O_2_peak at sea level (SL), 51.9 (±9.9) ml/min/kg]. Of the total sample, 21 (78%) had previously been to high altitude (>3,000 m), though nobody within the previous 3 months. For logistical reasons, participants were separated into two groups (A and B), according to their availability. Participants in both groups underwent sea level (SL) testing over two weekends (again, allocation to testing weekend was according to availability) and, approximately 1 month later, groups A and B ascended to the Margherita Hut [4,559 m, high altitude (HA)] 1 week apart. The study received institutional Ethical Approval from University College London and University of Turin. Prior to enrollment, all participants provided written informed consent and successfully completed a health screening process detailed previously (Martin et al., 2013).

### Setting and Ascent Profile

Baseline measurements were taken in London, England (75 m, day 0). Participants began the expedition by flying to Milan (102 m) where they stayed overnight. The following day, they traveled by road, ski lifts and on foot to the Gnifetti Hut (3,611 m). From this point, groups A and B had different ascent profiles due to a forecast of severe weather in the region altering Group A’s ascent profile. After 2 days at Gnifetti Hut, Group A ascended by foot to the Margherita Hut (4,559 m) where they stayed for testing for the remaining 8 days of the study. Group B stayed at the Gnifetti Hut for the scheduled 3 days, before trekking to the Margherita Hut, where they remained for 7 days. Testing began after 5 days of being at high altitude (HA) and continued for 3 days, designated as days 1, 2, and 3 in Supplementary Figure 1. To maintain an equivalent hypoxic “dose” within each group, participants remained within 300 vertical meters of the altitude of their overnight residence on non-ascent days.

### Intervention

This study used a randomized, double-blind, placebo-controlled factorial design, which has previously been described in full (Martin et al., 2013). Briefly, participants ingested either a beetroot/fruit juice beverage with high levels of nitrate [18.5 (±2.0) mmol] (BRJ) or a nitrate-depleted beetroot/fruit juice placebo [1.4 (±0.1) mmol] (PLA) control drink (produced and provided by AURAPA Würzungen GmbH, Bietigheim-Bissingen, Germany). Participants consumed the supplement each day in three 200 ml doses, resulting in a daily nitrate consumption of ∼0.18 mmol/kg/day and ∼11.5 μmoles/kg/day in the BRJ and PLA groups, respectively. Supplementation commenced 3 days prior to the exercise trials and continued throughout the testing period (see Supplementary Figure 1). During the study, food samples were taken from each meal and analyzed for their nitrate and nitrite content. The average daily consumption of nitrate and nitrite from meals was consistent with normal United Kingdom daily intake, with nitrate and nitrite intakes of 18 (±11) μmoles/kg/day and 0.038 (±0.023) μmoles/kg/day, respectively (Cumpstey et al., 2017). As reported previously, plasma nitrate concentration was approximately 4-fold higher in the nitrate supplementation group, compared to placebo [regression coefficient (95% CI); 1.5 (1.3, 1.7), *P* < 0.001)] and remained elevated throughout the duration of the exercise testing period both at sea level and at altitude (see Supplementary Table 1; Cumpstey et al., 2020). Inter-day variability in baseline circulating nitrate and nitrite concentrations was minimal in both groups as reported previously, enabling exercise testing to be performed over consecutive days at altitude (it was impossible to test all participants in such a large group on one single day due to time and logistical constraints) (Cumpstey et al., 2020).

### Exercise Testing

Participants underwent two exercise tests at SL and again at HA. The first test was a submaximal constant work rate test to determine exercise efficiency, and the second was a maximal exercise test investigating aerobic capacity. The tests were separated by at least 2 h of rest to allow time for recovery. Participants wore a facemask for measurement of breath-by-breath pulmonary gas exchange (Metamax 3B, Cortex, Leipzig, Germany) and cycled on an electromagnetically braked cycle ergometer (Lode Corival, Lode, Groningen, Netherlands).

To determine exercise efficiency, participants cycled at three constant work rates (20, 40, and 60 Watts) for 10 min each. The work rates were estimated to fall within the “moderate”-intensity exercise domain (i.e., below the ventilatory anaerobic threshold) to ensure estimates of efficiency were not affected by any increase in VO_2_ above AT (i.e., the VO_2_ slow component). *Post hoc* examination of ramp test data confirmed the work rates fell within the moderate intensity domain for all participants. Exercise efficiency was calculated from the final 5 min of expired air data from each exercise stage using a 4-step process. Firstly, interpolation was used to transform breath-by-breath data to 20 s average data. Secondly, data was screened to ensure V̇O_2_ did not increase more than 100 mls in the 5 min analysis window. This was the case for all tests. Thirdly, the mean value for VO_2_ and VCO_2_ were used to calculate energy expenditure using the equation of Brouwer (1957). Finally, external work performed on the ergometer and energy expenditure results were used to calculate delta efficiency using linear regression.

A symptom-limited, incremental ramp cycling protocol to volitional exhaustion was performed at SL and HA by each participant to determine V̇O_2_ peak and AT. The test began with 3 min of rest and a 3 min “unloaded” warm up, then participants performed the ramp section of the test to exhaustion. The work rate during the ramp increased by between 20 and 40 Watts each minute, depending on the fitness status of the participant and the altitude of the test. V̇O_2_ peak was defined as the average of the highest exertional oxygen uptake achieved over the last 20 s of exercise. The AT was determined using the modified V-slope method (Beaver et al., 1986), confirmed by patterns of change in ventilatory equivalent and end-tidal gas measurements (Whipp et al., 1986). Each test was independently analyzed by two assessors (authors: PH and AO’D), each trained and experienced in AT determination. When assessors selected an AT with a difference of less than 5%, the value selected for subsequent analysis was agreed through discussion (*n* = 51). For cases where assessors 1 and 2 disagreed by more than 5% (*n* = 3 tests), a third opinion was sought (author: DL) to resolve the discrepancy. This method of AT determination has been previously validated against arterial lactate threshold values measured at high altitude (Levett et al., unpublished data). In addition to expired air gas analysis, continuous heart rate measurements were made, blood pressure was taken every 3 min, and a 3-lead ECG was continuously monitored (Multilyser, Cortex, Leipzig, Germany).

### Plasma Collection and Biomarker Analysis

**FIGURE 1:**
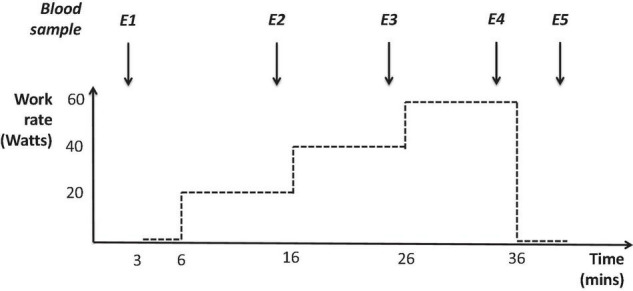
Profile of submaximal constant work rate exercise tests (performed at both sea level and high altitude) and the five time points where plasma was collected during these tests: the beginning of the test (E1), 2 min before the end of the 20 W, 40 W, and 60 W stages (E2, E3, and E4, respectively) and immediately before finishing the test during unloaded recovery (E5).

Full methods for plasma sampling and analysis have previously been detailed elsewhere (Cumpstey et al., 2020). Briefly, fasted venous blood samples (5 ml) were collected first thing in the morning at SL and on the 1st, 3rd and 5th testing mornings (D1, D3, and D5) during the study at HA, using EDTA-containing BD Vacutainer™ tubes. Samples were also collected at 5 time points during the submaximal constant work rate exercise tests (E1–5); at the beginning of the test (E1), 2 min before the end of the 20 W, 40 W, and 60 W stages (E2, E3, and E4, respectively) and immediately before finishing the test during unloaded recovery (E5), see Figure 1. Every sample was centrifuged at 800 × *g* for 15 min immediately after collection, aliquoted into separate cryovials and frozen (–40°C at the mountain and during transport, then –80°C until analysis).

All plasma biomarker concentrations were quantified after reaction with an excess of N-ethylmaleimide (10 mM NEM, in 10 mM phosphate buffered saline) added immediately after frozen plasma aliquots were thawed. To quantify plasma nitrite and nitrate concentrations, NEM-treated samples were deproteinized with methanol (1:1) and centrifuged at 16,100 × *g* for 10 min before undergoing analysis by high-pressure liquid chromatography (HPLC) using a dedicated nitrite/nitrate analyzer (ENO20, Eicom). All sample analysis was performed with repeated daily calibrations and staggered to ensure processing times were consistent, and all reported values were corrected for background levels of nitrite/nitrate. Nitroso product concentrations were quantified by group-specific denitrosation of NEM-treated EDTA plasma samples after injecting samples incubated with acidic sulfanilamide directly into an acidic triiodide-containing reaction chamber and measuring the NO liberated following reductive cleavage of protein nitroso-species by gas phase chemiluminescence (CLD 77am sp, EcoPhysics) as described (Feelisch et al., 2002).

### Data and Statistical Analysis

It was calculated that a sample size of 14 participants in each group would provide sufficient statistical power (0.8; β = 0.20) to detect a 10% difference in exercise efficiency during exercise from SL to HA using an alpha level of 0.05 (StatMate2, GraphPad Software, San Diego, CA, United States). These calculations were based on exercise efficiency being approximately 22.3 (± 1.8)% at sea level in healthy volunteers (Levett et al., 2011) and beetroot juice improving a related variable, O_2_ cost of exercise at sea level, by 7.1% (Larsen et al., 2007). We expected greater improvement in exercise efficiency in hypoxia due to a more active nitrate–nitrite–NO reduction pathway and thus opted to power the study to detect at 10% difference. Initially, 28 participants (14 in each group) were recruited, but one individual was unable to complete testing at high altitude due to altitude related sickness [detailed in Martin et al. (2013) and Cumpstey et al. (2017)], leaving data from 27 participants available for analysis.

Linear mixed modeling was used to analyze data to account for repeated measures and the different ascent profiles of each trek group (STATA 11; StataCorp LLC, College Station, TX, United States). All possible main effects and interactions of each outcome variable were compared across the two experimental groups, two altitudes, and two trek group accent profiles. The interaction between the various independent variables did not improve the fit of any of the models and therefore were not presented. Coefficients and *p*-values are provided for; the treatment effect in response to taking the high nitrate dietary supplement, the effect of altitude, and the effect of the different trekking group ascent profile. Normally distributed data are presented as mean and 95% confidence intervals (CIs) and non-normally distributed data as median and inter-quartile (IQ) range. *P*-values < 0.05 were considered significant.

## Results

### Exercise Efficiency

Delta exercise efficiency was not altered by altitude exposure or by nitrate supplementation (Table 1). Additionally, no interaction effect was present between altitude and nitrate supplementation.

**TABLE 1:** Descriptive results and regression coefficients for exercise physiological variables according to linear mixed modeling.

Altitude	Placebo	Treatment	Mixed model analysis	β	95% Conf. int.	*P*-value
**Delta Efficiency (%)**						
SL	26.3 (2.4)	26.5 (3.0)	Experimental group	0.138	−1.17, 1.44	0.836
			Altitude	−1.20	−2.50, 0.108	0.072
HA	24.4 (2.2)	26.1 (2.6)	Trek group	0.832	22.5, 27.5	0.227
**V̇O_2_peak (ml/kg/min)**						
SL	55.1 (8.4)	48.4 (10.5)	Experimental group	−5.90	−11.5, −0.227	0.041
			Altitude	−12.2	−14.2, −10.2	<0.001
HA	42.2 (6.5)	36.9 (8.0)	Trek group	3.14	−2.73, 9.00	0.295
Work rate max* (Watts)						
SL	328 (284, 336)	313 (224, 343)	Experimental group	−0.0783	−0.219, 0.0620	0.274
			Altitude	−0.200	−0.236, −0.166	<0.001
HA	261 (243, 313)	260 (224, 295)	Trek group	−0.052	−0.197, 0.0937	0.487
**Heart rate max (beat per minute)**						
SL	181 (9)	178 (10)	Experimental group	−2.57	−9.73, 4.59	0.482
			Altitude	−17.5	−23.1, 11.8	<0.001
HA	163 (12)	161 (16)	Trek group	3.51	−3.88, 10.9	0.352
**V̇e max (L/min)**						
SL	126 (22)	109 (33)	Experimental group	−18.8	−38.1, 0.572	0.057
			Altitude	31.7	24.8, 38.7	<0.001
HA	159 (29)	139 (30)	Trek group	−4.42	−24.4, 15.6	0.665
V̇O_2_ at AT* (ml/kg/min)						
SL	27.5 (22.4, 31.2)	26.1 (23.0, 29.9)	Experimental group	−0.0672	−0.235, 0.100	0.431
			Altitude	−0.259	−0.325, −0.193	<0.001
HA	21.1 (18.1, 23.7)	21.4 (15.8, 24.8)	Trek group	0.0847	−0.0884, 0.258	0.338

SL, sea level; HA, high altitude; V̇O_2_peak, peak oxygen consumption; V̇e, minute ventilation; V̇O_2_, oxygen consumption; AT, ventilatory anaerobic threshold. Data are presented as mean (±SD) or median (IQR) as appropriate. *Mixed effect multiple linear regression analysis conducted with log transformed data.

### Peak Oxygen Consumption and Anaerobic Threshold

Peak oxygen consumption decreased by 24% for the whole group at HA (*P* < 0.001) (Table 1). V̇O_2_peak was lower in the group supplemented with dietary nitrate at both SL and HA compared to the placebo group (*p* = 0.041) (Table 1). However, no interaction effect was present between high altitude and nitrate supplementation.

Other peak exercise variables changed in response to altitude for the whole group; maximum work rate was reduced by 18% (*p* = 0.041), maximum heart rate was lowered by 11% (*p* = 0.041), and peak minute ventilation (V̇e) was increased by 21% (*p* = 0.041) (Table 1). Dietary nitrate supplementation did not significantly alter the peak values of these physiological variables (Table 1).

Oxygen consumption at AT was also lower at HA (23%), when compared to SL (*p* < 0.001), but was not altered by nitrate supplementation (*p* = 0.431) (Table 1).

### Plasma Biomarkers

**FIGURE 2:**
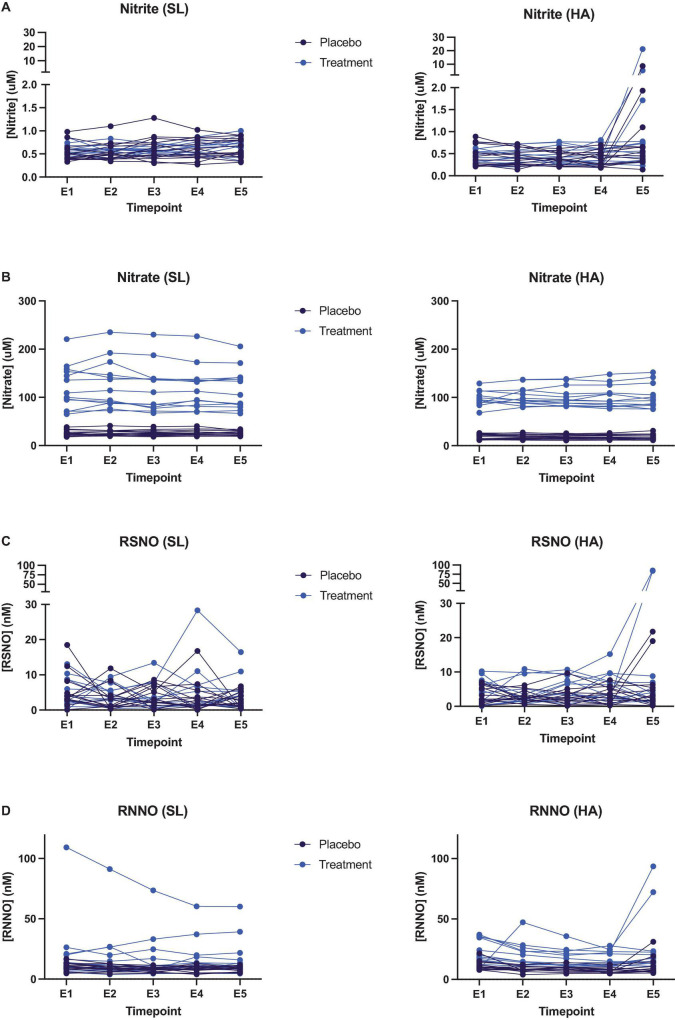
Quantification of plasma biomarkers collected at different timepoints during exercise efficiency tests at both sea level (left hand panels) and high altitude (right hand panels) in participants taking either the placebo (black data points) or high nitrate (blue data points) supplement. **(A)** Plasma nitrite; **(B)** plasma nitrate; **(C)** plasma S-nitrosothiols (RSNO); **(D)** plasma N-nitrosamines (RNNO).

At the time of exercise testing, circulating nitrate concentrations in individuals of the nitrate-supplemented group revealed a greater variability at SL compared to high altitude. We did not observe any significant change in nitrate, nitrite or nitroso product concentrations during the exercise efficiency tests, neither at SL nor at HA (see Figure 2 and Supplementary Table 2). In some individuals, circulating nitrite and nitroso product (particularly S-nitrosothiol) concentrations rose abruptly right after cessation of exercise in the efficiency testing protocol (timepoint E5 in Figure 2).

## Discussion

### Main Findings

Our finding that nitrate supplementation did not alter exercise efficiency at HA should be viewed in the context of a conflicting body of literature indicating that, following supplementation, submaximal oxygen utilization may either improve (Masschelein et al., 2012; Kelly et al., 2014; Muggeridge et al., 2014; Shannon et al., 2016, 2017) or remain unchanged (MacLeod et al., 2015; Horiuchi et al., 2017; Nybäck et al., 2017; Rossetti et al., 2017) under hypoxic conditions. Furthermore, neither AT, peak work rate, heart rate, nor ventilation were affected by nitrate supplementation. Meanwhile, V̇O_2_peak was lower following nitrate supplementation at SL and HA which supports some previous literature for hypoxic exposures at sea level (Kelly et al., 2014), but contradicts others (Arnold et al., 2015; Nybäck et al., 2017), though no interaction effect was present between altitude and nitrate supplementation. Overall, these findings suggest that supplementary nitrate is largely ineffective at altering exercise physiological responses to terrestrial high altitude.

No single variable appears to account for these conflicting results; with similar participant demographics, exercise test modalities and intensities revealing both positive and no effects of BRJ on exercise physiological responses to hypoxia. The lack of apparent effect cannot be attributed to ineffective supplementation either, as all studies that measured plasma nitrate and nitrite concentration found them to be elevated, irrespective of the study outcome (Masschelein et al., 2012; Kelly et al., 2014; Muggeridge et al., 2014; MacLeod et al., 2015; Shannon et al., 2016, 2017; Horiuchi et al., 2017; Nybäck et al., 2017; Rossetti et al., 2017).

In this study, plasma nitrate was four-fold higher in the nitrate supplementation group (regression coefficient (95% CI); 1.5 (1.3, 1.7), *P* < 0.001) (Cumpstey et al., 2020), yet increasing work rate did not significantly alter plasma concentrations of nitrite, nitrate or nitroso species. Although, plasma nitrite and nitroso species did increase in a number of cases (often but not universally in the supplemented group) immediately after exercise ceased. This phenomenon may be related to the utilization of nitrite and nitroso species during exercise. Conceivably, ongoing sequential conversion of nitrate to nitrite and nitrite to nitroso products and NO in skeletal muscle can give rise to increased translocation of intermediary products from muscle to the systemic circulation upon abrupt cessation of the metabolic hypoxic stimulus. Translocation of nitrite and nitroso species may be triggered by the sudden change in oxygen supply and demand as contractile activity stops. Little is known about the utilization of nitroso products during exercise, but the conversion of nitrite to nitroso species and NO (by various distinct mechanisms) is facilitated in hypoxia (Lundberg et al., 2009) and oxygen inhibits metabolic conversion of nitrite to NO (Feelisch et al., 2008). Thus, the rapid elevation of tissue oxygen availability in muscle may act as a break on both the downstream utilization of nitroso products and nitrite to NO reduction, with subsequent release of accumulating nitrite and nitroso species into lymph and blood. Why this process should occur faster in some individuals than others is not immediately obvious and warrants further investigation.

Skeletal muscle represents a quantitatively significant site of nitrate storage (Piknova et al., 2015), and its uptake from the circulation is complex (Gilliard et al., 2018; Wylie et al., 2019). Nitrate has been proposed to act as a regulator of systemic NO homeostasis by conversion into other NO-related species (Piknova et al., 2022) and may thereby confer protection against tissue damage. NO plays a key role in enabling oxygen and nutrient delivery by improving blood flow, and also in matching energy supply with demand by modulating mitochondrial function and intermediary metabolism (Umbrello et al., 2013). The variability in quality and magnitude of the nitrite and nitrosothiol accumulation observed after cessation of exercise suggests differences in metabolic fluxes exist between individuals, however, the significance of this observation is currently unclear and warrants further investigation. Stable-isotope labeled nitrate studies could characterize the role of individual nitrate-related metabolites in these pathways.

The lack of differences observed for most variables in this study could be related to the supplementation regime. In most previous studies, a single high dose (bolus) of nitrate (∼6–13 mmoles) was consumed 2–3 h prior to the exercise trial (Kelly et al., 2014; Muggeridge et al., 2014; MacLeod et al., 2015; Carriker et al., 2016; Shannon et al., 2016, 2017; Nybäck et al., 2017; Rossetti et al., 2017). Whereas, our ∼8.5 mmol daily dose was split into 3 equal parts consumed throughout the day, designed to produce a sustained elevation in plasma nitrate. Consuming a single high dose (8.4 mmol) of nitrate increases plasma nitrate to ∼300 μM after 2 h (Wylie et al., 2013), whereas average plasma nitrate concentration in the intervention group of the current study was ∼85 μM. As we have discussed previously, these lower plasma nitrate values may also reflect a hypoxia-mediated loss of plasma nitrate due to increased nitrate utilization, uptake by other tissues, or elimination from the body (Cumpstey et al., 2020). Such changes would be unlikely to occur during very short-lived hypoxic exposures, possibly explaining the higher plasma nitrate values observed in acute studies. Circulating plasma nitrate levels correlate with administered dose (Wylie et al., 2013). If peak nitrate concentrations, rather than its sustained elevation over time, drives nitrate induced physiological changes then this may explain why differences were not observed here. Despite a significant elevation of circulating nitrite at day 5, circulating nitrite concentrations were not significantly different from those in the placebo group on all other days (see Supplementary Table 1; Cumpstey et al., 2020). If nitrite (rather than nitrate) is the driver for alterations in exercise physiology, then this is the most likely explanation for why no such changes were observed in this study.

However, nitrate supplementation was not ineffective overall. Besides the elevation in exhaled NO reported previously (Cumpstey et al., 2017), one variable that was clearly altered by nitrate supplementation in the present study was V̇O_2_peak, which was lower in the treatment group at both SL and HA. A nitrate-induced reduction in V̇O_2_peak has previously been reported at SL (Larsen et al., 2007; Bescós et al., 2012) and in hypoxia (Kelly et al., 2014), though conflicting evidence also exists (Masschelein et al., 2012; Arnold et al., 2015; Perez et al., 2019). As no interaction was found between supplementation and altitude, either; (a) nitrate reduced V̇O_2_peak at SL and this reduction was sustained whilst at altitude, or (b) the random allocation of participants within the groups meant that this difference at sea level was a random error. The reduced V̇O_2_peak may be a reflection of alteration in electron flow within the mitochondrial respiratory chain but is unlikely to confer an advantage for exercise performance, except for possibly preventing tissue damage due to enhanced reactive oxygen species production at higher work rates. As this is the first study to investigate the effect of BRJ on V̇O_2_peak over this duration of altitude exposure, further study is warranted, particularly as no pre-supplementation measures were taken to rule out bias caused by potential issues of imperfect randomization of a relatively small sample.

Whilst similarities exist between the current study and previous literature on this topic, our results stand alone in that, for the first time, they show supplementary nitrate is not an effective ergogenic aid during prolonged periods (8 days) at terrestrial altitude. This may indicate that the potential benefits of supplementary nitrate on exercise outcomes some studies report are limited to acute hypoxia at sea level (Masschelein et al., 2012; Kelly et al., 2014; Muggeridge et al., 2014; Shannon et al., 2016, 2017). Previous results from this expedition also found nitrate was ineffective at altering resting respiratory function, blood pressure (Cumpstey et al., 2017), and microcirculatory flow (Cumpstey et al., 2020), and results from an earlier expedition indicated that BRJ also did not alter acute mountain sickness or basic physiological responses during an 11 days high altitude trek (Hennis et al., 2016). In contrast, acute BRJ supplementation has been reported to normalize brachial flow-mediated dilation after 7–8 days of high altitude, but was without effect on other physiological responses such as arterial oxygen saturation, vascular function and arterial blood pressure (Bakker et al., 2015). As hypoxia is typically experienced over prolonged periods, together, these results question whether nitrate supplementation has any practical utility at high altitude. However, in the absence of large numbers of studies conducted over this time frame the role of the supplement over this longer period is still largely unknown.

### Strengths and Limitations

This is the first investigation to study the effect of BRJ supplementation on exercise physiological responses during prolonged exposure to terrestrial altitude. This use of terrestrial altitude, rather than normobaric hypoxia, is important as physiological responses may differ between the two (Coppel et al., 2015). Furthermore, investigating the effectiveness of BRJ over multiple days at terrestrial high altitude more closely mimics how most people experience hypoxia, and thus results are more ecologically valid. The current study suffers from a number of limitations related to study design. Firstly, we used case-control rather than cross-over research design which introduces between-subject variation. The duration of altitude exposure precluded a cross-over design without including a prolonged (several months) “wash-out” period between two identical treks, which was not feasible. The increase in variability was combated by employing a sufficiently large sample size based on power calculations. Secondly, initial testing was preceded by 3 days of BRJ supplementation which precluded pre-supplementation testing without implementing an additional day of testing which was not possible due to time and logistical restraints. The omission of pre-supplementation measurements made it impossible to investigate whether variation in the physiological responses to BRJ ingestion could be attributed to differences in individuals’ capacity to increase circulating nitrate/nitrite, as has been previously suggested (Wilkerson et al., 2012). Finally, plasma nitrate concentrations provide a reserve for NO synthesis, the increases in plasma nitrate with BRJ that we observed were lower than acute studies that showed improvements in exercise variables in acute hypoxic environments. As such, much larger nitrate doses may be required to elicit beneficial effects on exercise variables at terrestrial altitude. Lastly, we cannot exclude that other biologically active BRJ constituents (Torrens and Feelisch, 2020) may have had confounding effects as they could have affected skeletal muscle physiology in the placebo group.

## Conclusion

This study indicates that, whilst consumed during an 8 days expedition to terrestrial high altitude, nitrate supplementation did not alter exercise efficiency. Furthermore, AT, peak work rate, heart rate, and ventilation were not affected by nitrate supplementation. V̇O_2_peak was lower in the group supplemented with dietary nitrate at both SL and high altitude. The results of this study and others question the practical utility of supplementing with BRJ during prolonged altitude exposure. However, this study is the first to investigate the role of BRJ on exercise physiological outcomes over a sustained exposure to terrestrial altitude, and thus further research is required before making definitive conclusions.

## Members of the Xtreme Alps Research Group

The Xtreme Alps research group members all contributed to the data collection for this study. The group consists of: Adam Booth, Adam Sheperdigian, Alasdair O’Doherty, Alex Salam, Alexandra Cobb, Andrew Cumpstey, Bernadette Fernandez, Damian Mole, Daniel Grant, Daniel Martin, Denny Levett, Edith Kortekaas, Edward Gilbert, Fabio Rigat, Fiona Shrubb, Heng Yow, James Farrant, Jildou van der Kaaij, Jim Milledge, Jo Simpson, Kay Mitchell, Laura Jackson, Liesl Wandrag, Lindsay Bisiker, Mark Edsell, Martin Feelisch, Maryam Khosravi, Matt Sanborn, Michael Grocott, Michael Mythen, Nick Talbot, Oliver Burdall, Oliver Firth, Oliver Moses, Paula Meale, Phil Hennis, Savini Wijesingha, Steve Dauncey, Tom Adams, Tom Woolley, Wilby Williamson, Will Jenner, and Zeyn Mahomed.

## Data Availability Statement

The original contributions presented in the study are included in the article/Supplementary Material, further inquiries can be directed to the corresponding author/s.

## Ethics Statement

The studies involving human participants were reviewed and approved by the University College London and University of Turin. The patients/participants provided their written informed consent to participate in this study.

## Author Contributions

HP, MM, MG, DL, DM, and MF: conception of the study. PH, AO’D, KM, ACo, PM, and BF: data collection. PH, ACu, AO’D, BF, and HM: data analysis. PH, ACu, and MF: writing of the manuscript. PH, ACu, MG, DL, DM, MF, and AO’D: editing of the manuscript. All authors: final approval of manuscript.

## Conflict of Interest

MG serves on the medical advisory board of Sphere Medical Ltd. and is a director of Oxygen Control Systems Ltd., received honoraria for speaking for and/or travel expenses from BOC Medical (Linde Group), Edwards Lifesciences, and Cortex GmBH, leads the Xtreme-Everest Oxygen Research Consortium and the Fit-4-Surgery research collaboration, and serves as the UK NIHR CRN national specialty group lead for Anaesthesia, Perioperative Medicine and Pain and is an elected council member of the Royal College of Anaesthetists. DM has received lecture and consultancy fees from Siemens Healthineers and Edwards Lifesciences. MM is a paid Consultant for Edwards Lifesciences, his University Chair was sponsored by Smiths Medical, founding Editor of the Journal of Perioperative Medicine and sits on the Editorial Board of the British Journal of Anaesthesia, and Editor-in-Chief of TopMedTalk. HP was employed by the company AURAPA Würzungen GmbH. The remaining authors declare that the research was conducted in the absence of any commercial or financial relationships that could be construed as a potential conflict of interest.

## Publisher’s Note

All claims expressed in this article are solely those of the authors and do not necessarily represent those of their affiliated organizations, or those of the publisher, the editors and the reviewers. Any product that may be evaluated in this article, or claim that may be made by its manufacturer, is not guaranteed or endorsed by the publisher.
