# Supplementary material for: Dietary Nitrate Supplementation Does Not Alter Exercise Efficiency at High Altitude – Further Results From the Xtreme Alps Study
Source: Front Physiol. 2022 Feb 28;13:827235. doi: 10.3389/fphys.2022.827235 (PMC8918982; doi:10.3389/fphys.2022.827235)
Supplement: Supplementary file 1 [file Data_Sheet_1.docx]

Data Supplement

to

Dietary nitrate supplementation does not alter exercise efficiency at high altitude – Further results from the Xtreme Alps study

Philip J Hennis^1, 2†^, Andrew F Cumpstey^3, 4†^, Alasdair F O’Doherty^5^, Bernadette O Fernandez^4,6^ Edward T Gilbert-Kawai^1^, Kay Mitchell^3, 4^, Helen Moyses^3^, Alexandra Cobb^1^, Paula Meale^1^, Helmut Pöhnl^7^, Monty G Mythen^1^, Michael P W Grocott^3, 4^, Denny Z H Levett^3, 4^, Daniel S Martin^1,8*††^ and Martin Feelisch^3, 4, 6*††^ and the Xtreme Alps research group.

1. Centre for Altitude, Space and Extreme Environment (CASE) Medicine, University College London Hospital NIHR Biomedical Research Centre, Institute of Sport Exercise & Health, London, UK
2. SHAPE Research Group, School of Science and Technology, Nottingham Trent University,

Nottingham NG11 8NS, UK

1. Perioperative and Critical Care Theme, NIHR Southampton Biomedical Research Centre, University Hospital Southampton NHS Foundation Trust, Southampton, UK
2. Integrative Physiology and Critical Illness Group, Clinical and Experimental Sciences, Faculty of Medicine, University of Southampton, Southampton, UK
3. Department of Sport, Exercise and Rehabilitation, Northumbria University, Newcastle upon Tyne, UK.
4. Warwick Medical School, Division of Metabolic and Vascular Health, University of Warwick, Coventry, UK
5. AURAPA, Bietigheim-Bissingen, Germany
6. Peninsula Medical School, University of Plymouth, Plymouth, UK

†, †† these authors contributed equally

*** Correspondence:**[daniel.martin@plymouth.ac.uk](mailto:daniel.martin@plymouth.ac.uk) or [m.feelisch@soton.ac.uk](mailto:m.feelisch@soton.ac.uk)

**Suppl. Figure 1:** Ascent profile used during the Xtreme Alps expedition; modified from reference (35). Trek 1 and Trek 2 are represented by solid and dotted lines, respectively. Each arrow represents a day of supplementation with either beetroot juice or placebo juice. Supplementation started 3 days prior to each testing period, represented by the shaded bars. SL = sea level testing, D1 – D5 = altitude testing days 1 to 5.

**Suppl. Table 1:** Plasma nitrate and nitrite levels at sea level (SL) and on the 1^st^ (D1), 3^rd^ (D3) and 5^th^ (D5) testing days at high altitude (HA, 4559m). Data are presented as median (IQR).

| **Location** | **Placebo** | **Treatment** |
| --- | --- | --- |
| **Plasma Nitrate (μM)** |  |  |
| *SL* | 19.2 (16.0 – 22.3) | 76.9 (68.3 – 110.6) |
| *HA (day 1 or D1)* | 20.9 (17.3 – 22.8) | 85.7 (70.5 – 102.0) |
| *HA (day 3 or D3)* | 19.9 (16.9 – 22.9) | 83.0 (74.2 – 95.2) |
| *HA (day 5 or D5)* | 22.9 (22.0 – 26.4) | 84.3 (66.9 – 101.0) |
| **Plasma Nitrite (μM)** |  |  |
| *SL* | 0.779 (0.682 – 0.863) | 0.856 (0.623 – 1.594) |
| *HA (day 1 or D1)* | 0.332 (0.205 – 0.387) | 0.355 (0.303 – 0.451) |
| *HA (day 3 or D3)* | 0.311 (0.228 – 0.387) | 0.378 (0.301 – 0.448) |
| *HA (day 5 or D5)* | 0.312 (0.270 – 0.343) | 0.409 (0.354 – 0.447) |

Data previously published by Cumpstey et al. 2020 (36)

**Suppl. Table 2**: Plasma nitrate and nitrite levels during constant load cycle tests at sea level and testing days at the high altitude (4559m). E1 = the beginning of the test (E1), E2/E3/E4 = two minutes before the end of the 20 W, 40 W & 60 W stages respectively, E5 = immediately before finishing the test during unloaded recovery. Data are presented as median (IQR) and p values refer to differences between placebo and treatment groups (conducted on log transformed data using linear mixed modelling).

|  | **Placebo** | | **Treatment** | | **P Value** |
| --- | --- | --- | --- | --- | --- |
|  | **Sea level** | **High altitude** | **Sea level** | **High altitude** |  |
| **Nitrite** |  |  |  |  |  |
| *E1* | 0.45 (0.40 - 0.59) | 0.40 (0.25 - 0.69) | 0.56 (0.50 - 0.61) | 0.41 (0.29 - 0.54) | 0.36 |
| *E2* | 0.50 (0.40 - 0.63) | 0.35 (0.26 - 0.53) | 0.60 (0.54 - 0.62) | 0.45 (0.30 - 0.56) |  |
| *E3* | 0.53 (0.42 - 0.73) | 0.38 (0.25 - 0.50) | 0.59 (0.54 - 0.65) | 0.41 (0.30 - 0.58) |  |
| *E4* | 0.56 (0.43 - 0.77) | 0.38 (0.22 - 0.52) | 0.64 (0.53 - 0.68) | 0.42 (0.28-0.59) |  |
| *E5* | 0.54 (0.46 - 0.80) | 0.54 (0.34 - 0.66) | 0.67 (0.48 - 0.76) | 0.56 (0.36 - 0.78) |  |
| **Nitrate** |  |  |  |  |  |
| *E1* | 24.05 (22.6 - 27.31) | 20.14 (13.37 - 23.93) | 109.18 (94.84 - 153.37) | 95.12 (87.78 - 103.96) | <0.0001 |
| *E2* | 24.58 (21.48 - 28.90) | 17.21 (14.19 - 21.64) | 114.39 (88.57 - 146.71) | 96.29 (89.36 - 114.51) |  |
| *E3* | 25.46 (22.05 - 29.12) | 17.62 (15.57 - 21.92) | 110.58 (79.65 - 138.57) | 93.55 (88.16 - 107.19) |  |
| *E4* | 24.26 (20.22 - 27.58) | 16.99 (14.57 - 21.87) | 112.71 (83.84 - 135.57) | 92.18 (84.24 - 109.36) |  |
| *E5* | 23.88 (21.16 - 28.29) | 18.37 (15.15 - 21.96) | 105.14 (84.25 - 140.79) | 95.96 (85.17 - 129.87) |  |
| **RSNO** |  |  |  |  |  |
| *E1* | 3.90 (3.06 - 4.80) | 2.64 (1.31 - 6.05) | 2.90 (2.20 - 8.65) | 4.61 (1.43 - 7.52) | 0.056 |
| *E2* | 2.19 (0.86 - 3.54) | 2.51 (1.57 - 3.37) | 3.32 (2.78 - 5.58) | 2.01 (1.64 - 3.89) |  |
| *E3* | 3.04 (2.01 - 6.26) | 2.34 (1.18 - 3.96) | 2.08 (1.47 - 3.60) | 4.14 (2.80 - 7.53) |  |
| *E4* | 2.27 (1.07 - 3.67) | 3.35 (0.88 - 5.14) | 1.89 (1.24 - 6.51) | 3.50 (2.20 - 7.22) |  |
| *E5* | 1.96 (0.80 - 3.78) | 2.92 (1.54 - 5.39) | 4.13 (1.16 - 5.29) | 3.71 (1.45 - 6.95) |  |
| **RNNO** |  |  |  |  |  |
| *E1* | 10.78 (8.51 - 12.85) | 10.37 (9.11 - 12.45) | 13.62 (9.60 - 20.12) | 18.61 (15.34 - 34.62) | 0.049 |
| *E2* | 9.09 (7.93 - 10.76) | 8.09 (7.41 - 10.15) | 10.66 (7.36 - 19.89) | 17.56 (12.09 - 24.28) |  |
| *E3* | 8.79 (6.61 - 9.43) | 9.05 (7.62 - 10.03) | 8.90 (7.63 - 17.05) | 13.46 (11.38 - 21.17) |  |
| *E4* | 8.68 (7.35 - 10.61) | 6.59 (5.80 - 8.21) | 12.06 (8.31 - 18.42) | 14.21 (11.67 - 22.28) |  |
| *E5* | 8.94 (7.41 - 9.98) | 7.22 (6.49 - 10.63) | 11.56 (8.92 -15.85) | 17.52 (13.30 - 22.58) |  |
